# Resolving responsibility gaps for lethal autonomous weapon systems

**DOI:** 10.3389/fdata.2022.1038507

**Published:** 2022-12-06

**Authors:** Patrick Taylor Smith

**Affiliations:** Stockdale Center for Ethical Leadership, United States Naval Academy, Annapolis, MD, United States

**Keywords:** lethal autonomous weapon systems, ethics of artificial intelligence, military ethics, responsibility, responsibility gaps, ethics of technology

## Abstract

This paper offers a novel understanding of collective responsibility for AI outcomes that can help resolve the “problem of many hands” and “responsibility gaps” when it comes to AI failure, especially in the context of lethal autonomous weapon systems.

## Introduction

This paper provides the normative grounding and a general description of a political conception of responsibility for just war compliance and non-compliance by lethal autonomous weapon systems. Deploying the Unfair Burden Argument, the Agent Constitution Argument, and the Collective Values Argument, the paper shows that we should move away from an interpersonal and ethical understanding of responsibility to a collective and holistic distributive conception of responsibility where we assign various accountability mechanisms and responsibilities to agents in the system on the basis of effectiveness and fairness rather than direct moral responsibility. This new account dissolves the problem of responsibility gaps for lethal autonomous weapon systems and points a way forward toward appropriately and legitimately distributing responsibility through the defense statecraft ecosystem.

## Research article

### The problem of responsibility gaps for lethal autonomous weapon systems

Lethal autonomous weapon systems (henceforth LAWS) are a set of proposed and speculative systems—though increasingly plausible—that mediate between human agency and the use of lethal force. Unlike automated systems that can fire on their own, autonomous weapon systems have the capability to function independently in a chaotic battlespace with little proactive human intervention (Horowitz and Sharre, 2015), operating along the kill chain without full human supervision. That is, they can use their own sensors and algorithms to acquire their targets and “decide” to fire on their own without a human decision. Such systems are obviously controversial and many militaries have rejected their use, but they have considerable potential utility, especially in cases where human reactions will be too slow or where communications between operator and drone are disrupted. Since LAWS are meant to operate on their own, they can operate unpredictably and act in ways that no individual operator or programmer would endorse. We can imagine cases where every human agent does what they can to reasonably foresee potential failure points and the unpredictable nature of the interaction between system and environment leads the LAWS to engage in an indiscriminate or disproportionate attack that violate the standard strictures of *jus in bello*. These actions could potentially involve the deliberate killing of non-combatant or the use of disproportionate force in a way that is unjust and immoral (Walzer, 2000).

As a consequence of this chaotic and unpredictable autonomy and the corresponding likelihood of just war violations, LAWS will almost certainly be involved in “responsibility gaps” (Sparrow, 2007; Asaro, 2012; Santoni and van den Hoven, 2018) where the system does something immoral and yet no person can be held accountable. Thus, LAWS might problematically “off-shore” potential responsibility by having the LAWs “make decisions” where it is genuinely unclear if anyone is truly responsible for the violation. There appears to be something uniquely bad about a non-human entity "making the decision” to violate the requirements of just war without any possibility of holding the violator responsible for the violation. Thus, the ultimate permissibility or impermissibility of LAWS may depend on finding a way to resolve the responsibility gaps inherent in its operation. Of course, there may be other reasons for rejecting unmanned, autonomous weapons (Emery and Brunstetter, 2015), but this paper is focused on evaluating and responding to the concerns generated by responsibility gaps.

Nonetheless, the notion of “responsibility” whereby the LAWS generates responsibility gaps is individualist (Smith, 2018). On this view, we need to develop an elaborate account of interpersonal ethics that tells us exactly the nature of our moral contribution and our level of responsibility for a particular set of decisions or consequences. Our obligations are then derived from this rigorous understanding of our responsibility. This generates the problem of responsibility gaps since it is not obvious that any one person can be assigned the relevant interpersonal moral status. But as we shall see, there is a different and more political way to understand responsibility: agents are responsible for what they would agree to under fair decision-making conditions (Simpson and Mullers, 2016). So, this paper takes the responsibility gap problem seriously but argues that it can be resolved if we understand responsibility differently in the military context.

### Rawlsian institutionalism and distributed responsibility

In what follows, I argue that we should adopt a “division of labor” whereby institutions *assign* individual responsibility much like institutions provide individuals with distributive shares. On this view, a person is responsible when an institutional *fairly* ascribes responsibility to the agent. Following a broadly Rawlsian understanding of the institutional division of labor in distributive justice (Rawls, 1971, 2001, and Pogge, 2000) an institutional division of labor is justified under three conditions (Smith, 2022). I will explain this idea in greater detail later, but first I will justify *why* we should move to a more collective understanding of responsibility in the first place. Thus, distributed responsibility is not a replacement for individual responsibility, but rather a supplement to it when particular conditions obtain. It is a matter of focus: do we start with institutions and *derive* individual obligations, or do we start with individual obligations and treat institutions as instruments for meeting them? Often, we do the latter, but there are conditions when we should do the former. This institutional priority and focus is also what distinguishes my view from other “distributed responsibility” views (Galliot, 2015) that nonetheless still start with the individualist, interpersonal conception.

First, a division of labor between individual and institutional obligations can be justified when satisfying the principles of justice requires practical coordination or epistemic demands that are unreasonable or impossible for individuals acting unilaterally. Taking Rawls's (2001) example, imagine a Lockean understanding of distributive justice of the following kind: we begin from a position of rough equality and then engage in a series of voluntary transactions that are just when they leave “enough and as good” for others. Initially, it might be possible for each person to have sufficient information and be able to anticipate what others are doing such that they could be reasonably confident that their individual choices satisfied the view. Yet, in any sort of complex society, the informational and cognitive requirements of understanding whether one was leaving “enough and as good” would be enormous. It would be unreasonable to expect any particular agent to be able to reliably make those judgments in all distributively relevant contexts. Similarly, ensuring that each person has the resources to engage in the foregrounded voluntary transactions will require intense practical coordination in terms of how much to give, what to give, and who should give. The idea here is that our obligations are *entangled* and that there is no *a priori* answer between various coordinative equilibria. As a consequence, there simply is no correct answer about the appropriate individual obligations without some authoritative, coordinative mechanism to determine individual contributions. And even if there were an optimal equilibrium to be discovered, this would only add to the informational and calculative burdens of individual agents. So, institutionalism can be justified when individual satisfaction of the principles is made impossible, unfair, or unreasonable by informational or coordinative burdens. In other words, institutions—by which I mean structures that use general rules and norms to purposively coordinate and direct human behavior—are required in order to maintain the background conditions for individuals to make fair and voluntary choices in their day-to-day economic, social, and political interactions. Call this the *unfair burden argument* for institutionalism.

Yet, we still might claim that the institution should be trying to replicate the *ideal*, aggregative choices of individual agents rather than claim that individuals have fundamentally different obligations from institutions. If we thought that imposing ideal individual obligations on actual individuals was unfairly or unreasonably burdensome, we would still want a moral division of labor, but we might still think that institutionalism was just there to “help” individuals satisfy their individual obligations. But there is another set of reasons for an institutionalist focus. If institutions play an essential role in *creating* and *maintaining* the agential capacities, powers, and resources that make it possible for individuals to propose, discuss, and abide by reasonable principles of justice, then we would need principles of justice that apply to those institutions over and above that of individuals. Insofar as institutions play an essential role in constituting the agency of the individual actor and have a large influence over the choice structure presented to the agent, then principles of justice need to apply to the institutions themselves. Otherwise, we will be imposing obligations upon agents without understanding or regulating the core influences upon that agent. It would seem odd to argue that individuals need to bear considerable burdens when faced with certain choices and not normatively evaluate the profound influence that the government, the family, or the market has over whether and to what extent the agent will have the capacities or resources to engage with those requirements in the first place. Call this the *agential influence argument* for institutionalism.

The *agential influence argument* provides an indicator of when institutionalism is necessary: different institutions will produce different agents with different capacities, facing different choices and circumstances. The *unfair burden argument*, on the other hand, suggests that we assign distinctive responsibilities to institutions. Combined, they suggest a kind of moral primacy for institutions for at least *some* questions: being a virtuous agent will do little to guarantee compliance with the relevant principles and good institutions can permit agents to be more self-interested and still produce just outcomes. So, if these arguments apply to a normative domain, then we have good reason to adopt an institutionalist paradigm whereby institutions are regulated by the principles of justice and individuals have an obligation to support those institutions and follow their dictates.

Finally, institutionalism may be justified when there are distinctive political values that can only be expressed or instantiated by collective institutions. For example, if deliberative democracy makes it possible for citizens to engage in binding, collective decision-making and it is an important political value that I participate in decisions that affect my core interests, we might think that the institutions of democratic decision-making are necessary for everyone to engage in legitimate, coordinated action. Similarly, Kantians and neo-republicans (Pettit, 1997; Young, 2000; Stilz, 2011) both argue, though for somewhat different reasons, that rightful relations between persons can only be achieved if mediated through political institutions that provide guarantees of their freedom from the domination of others. However, since this freedom needs to be assured by something other than the individual virtue, it is impossible for an individual to bring about these values on their own. If we accept these accounts of political freedom, then we must be institutionalists about—at least—these values as it is only through institutions that they are possible. Call this the *collective values argument*.

### Applying Rawlsian distributed responsibility to LAWS

In this section, I do two things. First, I show that these three arguments for institutionalism apply to lethal autonomous weapon systems. Second, I then show how institutionalism might be applied to resolve responsibility gaps for LAWS.

Let's take each of the three main arguments in turn. First, *unfair burden*. The chaotic and unpredictable nature of AI driven technology, even when well-tested validated, combines with the chaotic and unpredictable nature of the battlespace to make it very difficult, if not impossible, for individuals to make reliable, effective judgments with enough speed to prevent just war non-compliance. The cognitive burden of managing drone-human teams under chaotic conditions and the consequent unfairness of applying full responsibility to the user or commander is one of the drivers of responsibility gaps in the first place. For example, imagine that a commander is operating a “centaur” human-drone hybrid where the drone uses an algorithm to determine whether a target is a lawful combatant. The drone is in the process of “clearing” a room and determining it is safe for humans to enter and makes a split-second judgment that a person in the room is a combatant and kills them. It is very unlikely that the commander of the drone, or any member of the team, will always be able to intervene in real time to evaluate whether the drone is correct and then intervene to stop it if it is mistake. First, the drone is using perceptual capacities—radar, lidar, and the like—that the commander cannot easily process and is using rapid calculations to aggregate that data much faster than a human can comprehend. Even if the algorithm was explainable, the process would go by too quickly for the commander to remain “in the loop.” It is unreasonable to expect them to be able to do so. Thus, it seems plausible—as others have argued (Hayry, 2020; Verdiesen et al., 2021), but without the political foundations of this piece—that we need a broader understanding of institutional responsibility in the face of these concerns.

Second, LAWS will invoke concerns about agential constitution because these technologies will shape the very agency of the humans who will be participating in human warfighting. First, they will affect perception as the autonomous drones will feed information back to human warfighters, perhaps in spectrums and in formats that humans themselves cannot even perceive. Thus, drones will become part of our agency just as eyeglasses and hearing aids have become part of our agency, and this trend will only increase as we develop close-knit centaur human-AI teams as humans will be able to “see” the battlefield in certain ways due to their drone counterparts. Further, humans will come to understand what they can “do” in terms of delivering fire and shaping the environment in terms of what their drones can do. A human commander will understand that “they” can clear a room without using deeply coercive measures using LAWS but will also come to feel as they have decreased capacity when those drones are unavailable, just as we feel a reduction in our own capacities once the wireless internet stops working. In other words, we shape our own capacities based on the expectation that tools and technologies will be able to take up the slack, such as we when we stop memorizing phone numbers because smart phones will store them for us. That means, our own cognitive and physical capacities are structured by what we expect our tools to be able to do. A focus on individual moral responsibility at the cost of institutional distributed responsibility will miss the ways doctrinal, design, and deployment choices will shape the vary ways that humans act and perceive.

Finally, there are a plethora of collective and political values that apply to military action. Just war theory—as well as international law—is structured by the normative demand for proper authority: so appropriate constitutional legitimacy is a key a feature of the right to go to war (Fabre, 2008; Galliot, 2015). The use of autonomous systems in the military context will require *trust*, which is a feature of the institutions themselves. When an individual warfighter uses a drone, they are trusting a complex set of institutions that engaged in design, testing, and validation and whether those processes are trustworthy is a collective value. Also, protecting the rights of non-combatants and civilians who are subject to the authority and coercive power of soldiers requires more than just that soldiers *individually* refrain from war crimes, the rights of civilians must also be *assured* by substantial accountability mechanisms that mitigate the arbitrary authority and power that military personnel can have over civilians. Finally, some have argued that the practice of atoning for military ethics violations must be collective as individual soldiers will not be able to go through the practice of apology and reparations for individual victims. In general, soldiers operate within a collective context where what they do reflects on the collectivity and what the collectivity does reflects upon them, both for good and ill. Many of these considerations apply to the military in general, but these issues are only exacerbated with LAWS.

So, let's grant that we need an institutionalist orientation for responsibility for LAWS compliance and non-compliance just war principles rather than an interpersonal one. It would take too much space to fully delineate how this would work in practice, but I will offer some preliminary comments. There are three elements of a Rawlsian distributed responsibility account: an account of what is to be distributed, an account of the institutions that work together to distribute the responsibility and produce the normatively relevant outcomes, and a process to choose fair principles of distribution. Let's take each in turn.

First, the account concerns the distribution of responsibility, but it is essential to see that we can pull apart the various ways we hold people accountable. We hold people responsible in many ways: criminal liability, civil liability, career-oriented costs and benefits, and social opprobrium, amongst others. There is no reason that a political system would distribute these various mechanisms uniformly; instead, we should *disaggregate accountability mechanisms*. Suppose we believe that both a LAWS designer and a commander who deploys a LAWS that violates the principles of just war should be held responsible for the failure. On an interpersonal view, we might think the question is “responsible or not?” but on the political view the question now becomes, “What *sorts* of responsibility should we distribute onto the various agents?” So, we might hold the corporation who designed the LAWS civilly liable to compensate the victims while holding the commander liable through the diminution of their career prospects while saving criminal liability for other agents and social opprobrium for yet others. Again, accountability mechanisms are disaggregated and then distributed throughout the system to produce good outcomes in a fair way. This is one way that my more political conception is different from other collective responsibility views: they treat “responsibility” as a monolithic notion rather than one that can be disaggregated.

Yet, how should accountability be distributed such that it is fair? A final determination is beyond the scope of this paper, but I would like to describe how a broadly Rawlsian-constructivist (James, 2005) account might proceed based on the *veil of ignorance*. First, we would understand the complex set of institutions that produce LAWS outcomes as a kind of cooperative endeavor: political oversight. design, testing, evaluation, validation, training, doctrine, and deployment all work together as a web-like system of systems to generate a contextual rate of just war compliance by the specific LAWS that is created and used. A Rawlsian-constructivist—not necessarily Rawls himself—understanding of this cooperative structure lends itself to the following question, “Given the need to generate the relevant ethical values, how would we distribute various accountability mechanisms if we did not know where in the cooperative system we might find ourselves?” In other words, who would we hold accountable and why if we were ignorant of how those decisions might come to apply to us? This is a way of modeling fair decision-making as it prevents one from biasing the distribution based upon their knowledge that they will be powerful agents in the system and focuses attention on the common good (Huang et al., 2019). If I knew I was going to be a high-ranking officer, politician, or corporate executive, I might design a system that shields me from accountability. Yet, this is far less attractive if I do not know if I will be the executive or a young lieutenant facing the decision to use the drone in combat; the veil of ignorance forces me to decide on principles and distributions for everyone on an equal basis because I could be anyone in the system.

A consequence of these two features—disaggregation and the veil of ignorance—of institutional responsibility is that accountability will be distributed far more widely and holistically than one might traditionally believe and that there should be consequences for failure up and down the chain of decision-making for LAWS outcomes. If I knew I might be a young lieutenant deciding whether to deploy LAWS and that I would be held at least partially accountable for what happens, then I would demand principles that assigned accountability to other agents to ensure that I was placed in a position to succeed and that I could trust the reliability of the system. So, responsibility would move beyond the military chain of command to include the civilian leadership making decisions on where to go war and why, the technology and defense contractors designing the system, and the defense bureaucracy making choices on training and doctrine. Of course, if one knew that there was the possibility of being held accountable for the choices of the tactical commander in the field, then a system where the tactical commander had no responsibility for what happens would also be unacceptable. What is needed is to balance the relevant claims of the stakeholders within the defense statecraft ecosystem and for that, we need a political conception of distributed responsibility.

I will end this paper with a brief anecdote. I have taught military ethics to both experienced officers and midshipmen still waiting on their commissions, and they are taught to take responsibility to prevent war crimes and atrocities. Yet, I have also been shown the computer simulations used by defense consultants to wargame tactical decision-making and, indirectly, to contribute to doctrine and procurement. The very tools my midshipmen will possess are, in part, determined by these simulations. Yet, these simulations include *no* provision for preventing civilian casualties; it is not that they are ignored, it is that civilians do not exist. The consultants take essentially no responsibility in ensuring that warfighters have the appropriate tools to achieve their objective within the context of the rules of war as just war principles are left to others. This is both unsurprising and perfectly rational in the context of the interpersonal model: their contribution is far too indirect to activate individual, personal responsibility. Yet, it is deeply unfair that individuals who are much more powerful and well-connected, who have the time and money to think carefully, are “off the hook” while the newly-minted lieutenant facing combat for the first time feels the full brunt of accountability. Of course, military officers receive special training and develop specific virtues to handle this sort stress and this is relevant to responsibility attributions, but having power and authority within the system is *also* relevant. And this is especially true when the battlespace becomes populated by objects as complex as LAWS. To resolve this problem, we must reorient our thinking in a political direction.

## Data availability statement

The original contributions presented in the study are included in the article/supplementary material, further inquiries can be directed to the corresponding authors.

## Author contributions

The author confirms being the sole contributor of this work and has approved it for publication.

## Funding

This work was funded by Peace Research Institute Oslo, Norway for the Open Source fee.

## Conflict of interest

The author declares that the research was conducted in the absence of any commercial or financial relationships that could be construed as a potential conflict of interest.

## Publisher's note

All claims expressed in this article are solely those of the authors and do not necessarily represent those of their affiliated organizations, or those of the publisher, the editors and the reviewers. Any product that may be evaluated in this article, or claim that may be made by its manufacturer, is not guaranteed or endorsed by the publisher.
